# Selenium-containing polysaccharide from *Spirulina platensis* alleviates Cd-induced toxicity in mice by inhibiting liver inflammation mediated by gut microbiota

**DOI:** 10.3389/fnut.2022.950062

**Published:** 2022-11-03

**Authors:** Ning Zhou, Hairong Long, Lian Yu, Xianghua Xia, Zhenjun Zhu, Xiaoling Liu

**Affiliations:** ^1^College of Light Industry and Food Engineering, Guangxi University, Nanning, China; ^2^Guangxi Botanical Garden of Medicinal Plants, Nanning, China; ^3^Department of Food Science and Engineering, College of Science and Engineering, Jinan University, Guangzhou, China

**Keywords:** gut microbiota, liver inflammation, Cd-induced toxicity, selenium polysaccharide, *Spirulina platensis*

## Abstract

Selenium-containing polysaccharide from *Spirulina platensis* (Se-SPP) has been demonstrated to help in inhibiting cadmium-induced injury in mice, but the underlying mechanism has not been determined. This study aimed to investigate the beneficial effects of Se-SPP on alleviating Cd-induced toxicity in mice by targeting liver inflammatory and gut microbiota. Se-SPP supplementation for 28 days in Cd-induced toxic mice significantly mitigated liver pathological damage and inflammation, which was correlated to the upregulation of antioxidant enzyme activity. Furthermore, Se-SPP effectively restored Cd-induced disruption of the intestinal barrier compared to model group, as indicated by the depletion of *Muribaculaceae* and the enrichment of *Ruminococcaceae*. Spearman's correlation analysis revealed that the Se-SPP-altered microbes were highly correlated with inflammation-related indexes in Cd-induced toxic mice. Noteworthily, the modulation of Se-SPP on the *Ruminococcaceae* population contributed to the improvement of Cd-induced inflammation-related diseases by downregulating the tumor necrosis factor-α (TNF-α) and interferon-γ (IFN-γ) in the liver. These findings suggested that Se-SPP may act as prebiotics for ameliorating Cd-induced toxicity in mice by inhibiting liver inflammation mediated by gut microbiota, and target-specific microbiota of Cd-induced inflammation-related diseases deserve further attention.

## Introduction

As environment pollution worsens, cadmium (Cd), a toxic heavy metal widely used in agriculture and industry, has been one of the most common environmental pollutants threatening public health (1, 2). Cd is present in abundance in water, soil, and crops and enters the human body mainly through the food chain. Given its long half-life and strong molecular toxic effects, it easily accumulates in living organisms, resulting in a broad range of adverse health effects in humans and animals (3). The World Health Organization (WHO) has listed Cd as the priority food contaminant for research (4, 5). The liver plays an important role in detoxification in living organisms, so it is the main target organ which is most affected by Cd through all exposure patterns. The degree of hepatotoxicity depends on the amount and duration of Cd exposure (6). Cd causes free radicals and reactive oxygen species (ROS) to induce oxidative stress after entering hepatocytes through the transport system, and then excessive intracellular ROS is attributed to cellular inflammation, apoptosis, and necrosis, which may be one of the main mechanisms of Cd-induced hepatoxicity (7, 8). Due to the advantages of high bioavailability and minimal toxicity, natural functional products have gradually become major research objects for the elimination of hepatoxicity associated with Cd exposure (2). As a novel type of functional polysaccharides, selenium polysaccharides possess noticeable antioxidant activity depending on its free radical scavenging ability. Simultaneously, studies have proven that selenium polysaccharides possess hepatoprotective activity for repairing various liver damage caused by oxidative stress (9).

The gut microbiota plays a key role in maintaining intestinal barrier integrity (10). The intestinal barrier is essential to limit the absorption of cadmium, and the disruption of it exacerbates the absorption of cadmium (11). With increasing attention to the gut–liver axis, a number of studies have revealed that gut microbiota is involved in the mechanisms of regulating liver damage. The bidirectional relationship between gut microbiota and the liver leads to an inseparable relationship between the health of the liver and intestines (12). Degradation of polysaccharides in the intestines has an effect on the gut microbiota and short-chain fatty acid (SCFA) productivity. Recently, various natural polysaccharides have been certified to protect host health by modulating the gut microbiota (13–15). Wu et al. investigated the anti-inflammatory effect of *Cyclocarya paliurus* polysaccharides (CP) on CCl_4_-induced mice, suggesting that CP ameliorated liver inflammation in mice by regulating the gut microbiota composition and increasing the concentration of SCFAs (16). However, there is no report on either the effects of selenium-containing polysaccharides on the gut microbiota, or the influence of selenium-containing polysaccharides on Cd-induced changes in the gut–liver axis. Our previous work demonstrated that a one-time gavage of 6.5 mg/kg body weight of cadmium chloride resulted in liver injury, and selenium-containing polysaccharides from *Spirulina platensis* (Se-SPP) treatment provided significant protection against Cd-induced toxicity, but its underlying mechanism has not been studied yet. We hypothesized that Se-SPP alleviated liver inflammation in Cd-induced toxic mice *via* the regulation of gut microbiota. Thus, this study aimed to investigate the beneficial effects of Se-SPP on alleviating Cd-induced toxicity in mice by targeting liver inflammatory and gut microbiota. In addition, the correlation between the gut microbiota and liver inflammation parameters was analyzed.

## Materials and methods

### Reagents and materials

The selenium-containing *Spirulina platensis* was cultivated and collected in Beihai, China, and the preparation and characterization of Se-SPP was carried out as previously reported (17). Cadmium chloride (CdCl_2_) were purchased from Macklin (Shanghai, China). Mouse TNF-α, IL-1β, IFN-γ, and IL-10 ELISA kits were purchased from Bioswamp (Wuhan, China). SOD, CAT, GSH, and MDA assay kits were purchased from Nanjing Jiancheng Biotechnology (Nanjing, China). All the other reagents used in the present study were of analytical grade.

### Experimental design

Totally, 32 healthy male C57BL/6J mice (age 7–8 weeks; weight 16–18 g) were purchased from Beijing Vital River Laboratory Animal Technology Co., Ltd. (Beijing China). All animal testing procedures were approved by the Animal Ethics Committee of the Guangxi Institute of Chinese Medicine & Pharmaceutical Science (No. 2020110202). All mice were housed under standard laboratory conditions (constant temperature 23 ± 1 °C, humidity 60 ± 2 % and 12-h light/dark cycle) and acclimatized for 7 days prior to experiments. In each cage, four mice were housed, with distilled water and normal diet (Beijing Keao Xieli Feed Co., Ltd.) provided *ad libitum*.

All healthy male C57BL/6J mice were randomly assigned into four groups: normal control (NC), model control (MC), positive control (PC, Na_2_SeO_3_: 0.1mg/kg/day), and Se-SPP (100 mg/kg/day) groups, with eight mice in each group. In this study, CdCl_2_ was utilized to establish Cd-induced toxic mice models (18), and Na_2_SeO_3_ was used as the positive drug. The animal experiment was conducted according to previous methods with some modifications (19, 20). Except to the NC group, other groups were intervened with CdCl_2_ (7.5 mg/kg/day) by gavage for 2 weeks. Meanwhile, after 1-h Cd exposure, the PC and Se-SPP groups were, respectively, administered with tested drugs throughout the experimental period, as mentioned earlier; the NC and MC groups were gavage-administered an equivalent quantity of distilled water in accordance with body weight (0.1 mL/10 g weight). The dosage of Na_2_SeO_3_ and Se-SPP was determined by pre-experiments.

After 28 days of treatment, all mice were fasted overnight and then euthanized. The whole blood samples were obtained and centrifuged at 3,500 × g for 15 min at 4 °C. The serum samples were stored at −80 °C for the determination of hepatic function biomarkers. The liver and intestinal tissues were excised and processed for the further assays. In addition, fresh feces of mice were collected in sterile tubes and also stored at −80 °C for gut microbiota analysis.

### Biochemical analysis and cytokine measurements

#### Determination of liver coefficient in mice

The final body weight of animals from each of the four groups were recorded before being killed. After killing, the liver tissues were rapidly removed from the abdominal cavity and weighed, and the liver coefficient was calculated according to the following formula (21).


(1) $$\begin{array}{l} \text{Liver coefficient }\left(\text{\%}\right)=\left(\frac{\text{liver weight}}{\text{body weight}}\right)\times 100 \end{array}$$

#### Determination of hepatic apoptosis in mice

The paraformaldehyde-fixed and paraffin-embedded liver sections were subjected to terminal deoxynucleotidyl transferase-mediated dUTP nick-end labeling (TUNEL) assays using a commercial TUNEL staining kit (Beyotime, Shanghai, China) according to the manufacturer's instruction. The stained sections were observed under an inverted microscope (eVOS, Thermo Fisher Scientific, USA) at × 20 magnification. The TUNEL-positive cells were quantified by counting the number of apoptotic cells in five randomly selected fields from each liver tissue section. Data were expressed as percentages of TUNEL-positive areas (22).

#### Determination of hepatic function biomarkers in serum

Blood samples were collected by extirpating the eyeballs and centrifuged at 3,500 × g for 15 min, 4 °C [Universal 320R, Hettich Zentrifugen, Germany) to obtain serum, of which the levels of hepatic function biomarkers (alanine transaminase (ALT), aspartate transaminase (AST), alkaline phosphatase (ALP), total bilirubin (T-Bil), and lactate dehydrogenase (LDH)] were measured using commercial kits (Mindray Bio-Medical Electronics Co., Ltd, Shenzhen, China) according to manual instructions (23).

#### Determination of the hepatic inflammatory markers in mice

The fresh liver tissues were homogenized in ice-cold PBS (pH 7.4) and then centrifuged at 12,000 rpm for 20 min at 4 °C. The supernatants were obtained for further biochemical analysis (24). The levels of tumor necrosis factor (TNF-α), interleukin-1 beta (IL-1β), interferon-γ (IFN-γ), and interleukin-10 (IL-10) were determined using enzyme-linked immunosorbent assay (ELISA) kits (Cat. Number: 202103, Bioswamp) according to the manufacturer's instructions using an ELISA reader (AMR-100, Aosheng, China) (25).

#### Determination of the hepatic antioxidant enzyme contents in mice

The levels of superoxide dismutase (SOD), catalase (CAT), glutathione (GSH), and malondialdehyde (MDA) were measured using the commercial assay kits as per manufacturer's instructions (Jiancheng Bioengineering Institute, Nanjing, China) (26, 27).

### Histological evaluation

The tissues were excised, fixed immediately in 4% paraformaldehyde solution for 24 h, and then embedded in paraffin. Sections of 5 μm thickness were stained with hematoxylin and eosin (H&E). The tissue pathological changes of C57BL/6J mice were observed by light microscopy (eVOS, Thermo Fisher Scientific, USA) at × 20 magnification. Totally, three fields of vision in each sample were collected at random (14).

### Western blot analysis

Western blot was carried out as previously mentioned (28). Claudin-4 antibody was purchased from Thermo Fisher Scientific (USA). Tight junction protein 1 (ZO-1) and glyceraldehyde-3-phosphate dehydrogenase (GAPDH) antibodies were purchased from Bioswamp (Wuhan, China). All antibodies were diluted at 1:1000 in 4% BSA/PBS. The band intensity was measured by a TANON GIS system (Shanghai, China). Protein expression was estimated through band intensity quantitation and normalized by GAPDH.

### Gut microbiota analysis

An E.Z.N.A.^®^ Stool DNA Kit (Omega, USA) was utilized to extract total bacterial DNA from fecal samples according to the manufacturer's instructions. The V3-V4 regions of 16S rDNA of bacterial DNA were amplified with specific primers (341F and 805R). The amplified products were purified with AMPure XT beads (Beckman Coulter Genomics, Danvers, MA, USA) and quantified by Qubit (Invitrogen, USA). The amplicon pools were prepared for sequencing, and the size and quantity of the amplicon library were assessed using an Agilent 2100 Bioanalyzer (Agilent, USA) and the Library Quantification Kit for Illumina (Kapa Biosciences, Woburn, MA, USA), respectively. The samples were sequenced on an Illumina NovaSeq PE250 platform according to the manufacturer's recommendations, provided by LC-Bio Technology Company (Hangzhou, Zhejiang, China). Microbiota sequencing data were deposited in the Genome Sequence Archive in National Genomics Data Center (GSA: CRA008374) that are publicly accessible at https://ngdc.cncb.ac.cn/gsa. Paired-end reads were merged using FLASH. Quality filtering on the raw reads were performed under specific filtering conditions to obtain the high-quality clean tags according to fqtrim (v0.94). Chimeric sequences were filtered using VSEARCH software (v2.3.4). After dereplication using DADA2, feature table and feature sequence were obtained. Feature abundance was normalized using the relative abundance of each sample through SILVA (release 132) classifier. β-Diversity was calculated by QIIME2, and the related graphs were drawn by R package. Blast was used for sequence alignment, and the feature sequences were annotated using SILVA database for each representative sequence. Other diagrams were drawn using the R package (v3.5.2).

### Statistical analysis

All statistical analyses were performed using GraphPad Prism 8.0.2 statistical software, and the data are expressed as mean ± standard deviation (SD). All data were subjected to one-way ANOVA, where *p* < *0.05* was considered statistically significant. Spearman's rho nonparametric correlations between the antioxidant-related indexes and inflammatory factors and between the gut microbiota and inflammatory factors were determined using the R package (v3.6.3).

## Results

### Se-SPP alleviates Cd-induced liver injury in mice

**Figure 1:**
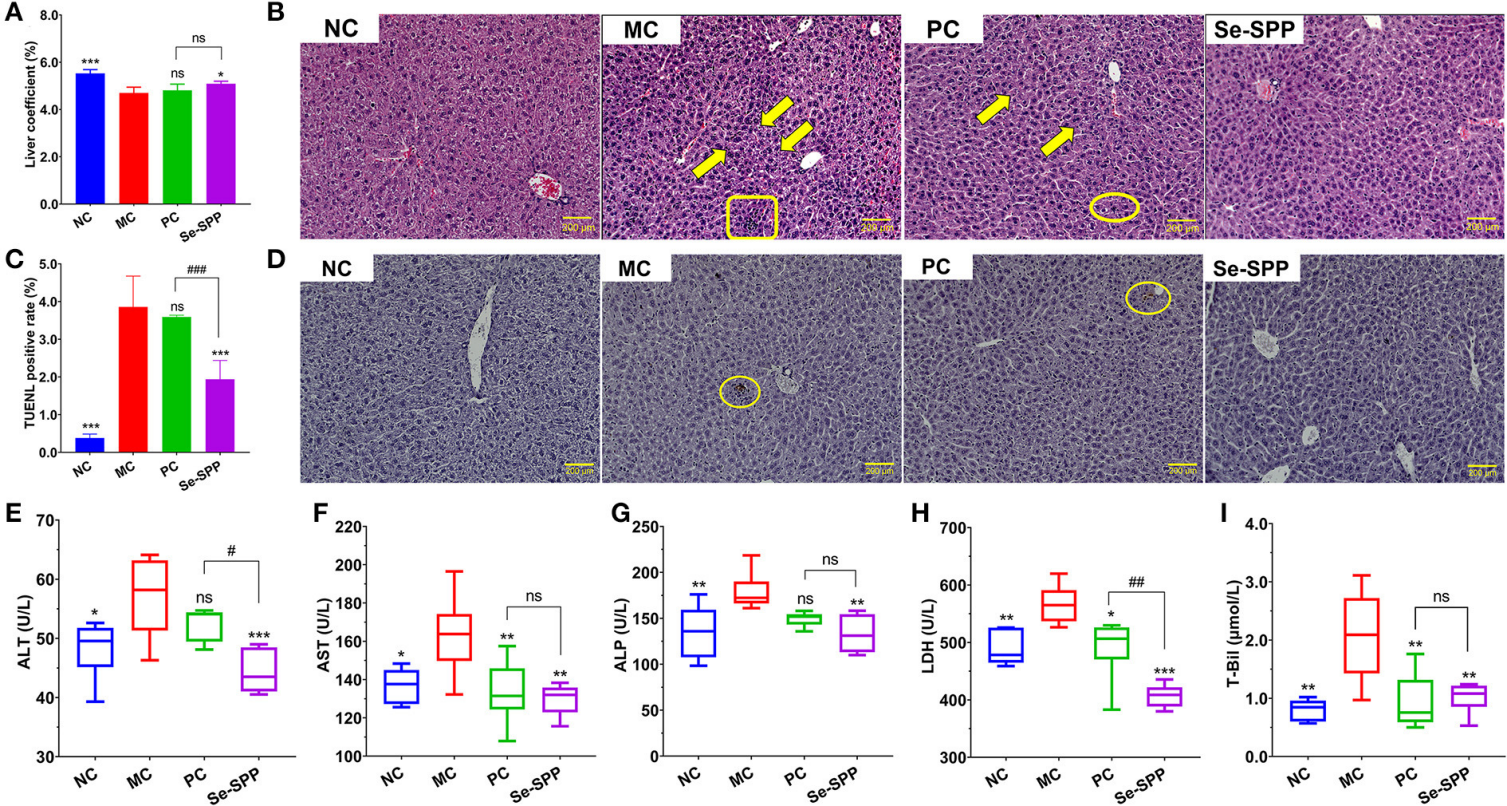
Se-SPP alleviates Cd-induced liver injury in mice. Liver coefficient **(A)**. Images of H&E-stained liver [**(B)**, 20×], karyopyknosis (arrow), punctate necrosis (circle), and inflammatory cell infiltration (rectangle). The TUNEL staining of hepatic cells **(C,D)** and serum T-Bil, ALT, AST, ALP, and LDH levels **(E–I)**. ALT, alanine transaminase; AST, aspartate aminotransferase; ALP, alkaline phosphatase; LDH, lactate dehydrogenase; T-Bil, total bilirubin; NC, normal control; MC, model control; PC, positive control; Se-SPP, selenium-containing polysaccharides from *Spirulina platensis*. Data are expressed as mean ± SEM (*n* = 6). Graph bars marked with ns: not significant, *p < 0.05, **p < 0.01, ***p < 0.001, when comparing with MC, ^#^*p* < 0.05, ^##^*p* < 0.01, ^###^*p* < 0.001 when comparing PC with Se-SPP.

The effect of Se-SPP on the liver coefficient of Cd-induced toxic mice is shown in Figure 1A. The liver coefficient in the MC group revealed an obvious decrease compared to the NC group (*p* < 0.001). After treatment, the liver coefficient was not significantly different between the MC and PC groups, while the liver coefficient in the Se-SPP group was significantly increased compared with that of the MC group (*p* < 0.05).

Histopathological examination directly reflected the pathological condition of liver tissue in the mice. The pathological sections in the liver of each group were observed under a × 20 microscope, as shown in Figure 1B. The liver tissues in the NC group showed a normal hepatic architecture with normal hepatic lobular pattern. However, CdCl_2_ treatments caused apparent pathological changes. The hepatic tissues in the MC group exhibited inflammatory cell infiltration, hepatocytes, karyopyknosis, and disordered hepatic cord. Both therapy groups indicated minor lesions compared with the MC group. The hepatic cords of PC and Se-SPP groups were arranged in order, and the percentage of hepatic cells with karyopyknosis significantly decreased. Likewise, a small number of erythrocytes was found in the hepatic sinus and central veins. In addition to the aforementioned symptoms, punctate necrosis was observed in the liver of the PC group.

The apoptotic nuclei stained brown through TUNEL staining and then detected (Figures 1C,D). The NC group presented few apoptotic hepatic cells. However, the percentage of TUNEL-positive cells in the liver expressed the proportion of hepatic apoptotic cells after CdCl_2_ treatment. The number of apoptotic hepatic cells in the MC group was noticeably increased when compared with the NC group, the difference was statistically significant (*p* < 0.001). The degree of apoptosis between the PC group and MC group was not statistically different (*p* > 0.05). By contrast, after the administration of Se-SPP, the number of apoptotic cells apparently decreased (*p* < 0.001) compared with the MC group.

To investigate the protective effect of Se-SPP on liver function in the Cd-induced model mice, the levels of the biochemical indicators of liver function in serum samples (T-Bil, ALT, AST, ALP, and LDH) were determined. As shown in Figures 1E–I, the levels of all the five biochemical indicators of liver function in the MC group were significantly higher than those in the NC group (*p* < *0.05* for ALT and AST, *p* < *0.01* for ALP T-Bil and LDH). By contrast, AST, T-Bil, and LDH levels of Na_2_SeO_3_-treated mice exhibited an apparent decrease compared with the MC group (*p* < *0.05* for LDH, *p* < *0.01* for AST and T-Bil). However, the contents of serum ALT, AST, ALP, T-Bil, and LDH downregulated dramatically *via* the intake of Se-SPP (*p* < *0.01* for AST, ALP, and T-Bil, *p* < *0.001* for ALT and LDH).

### Se-SPP alleviates Cd-induced liver inflammation in mice

**Figure 2:**
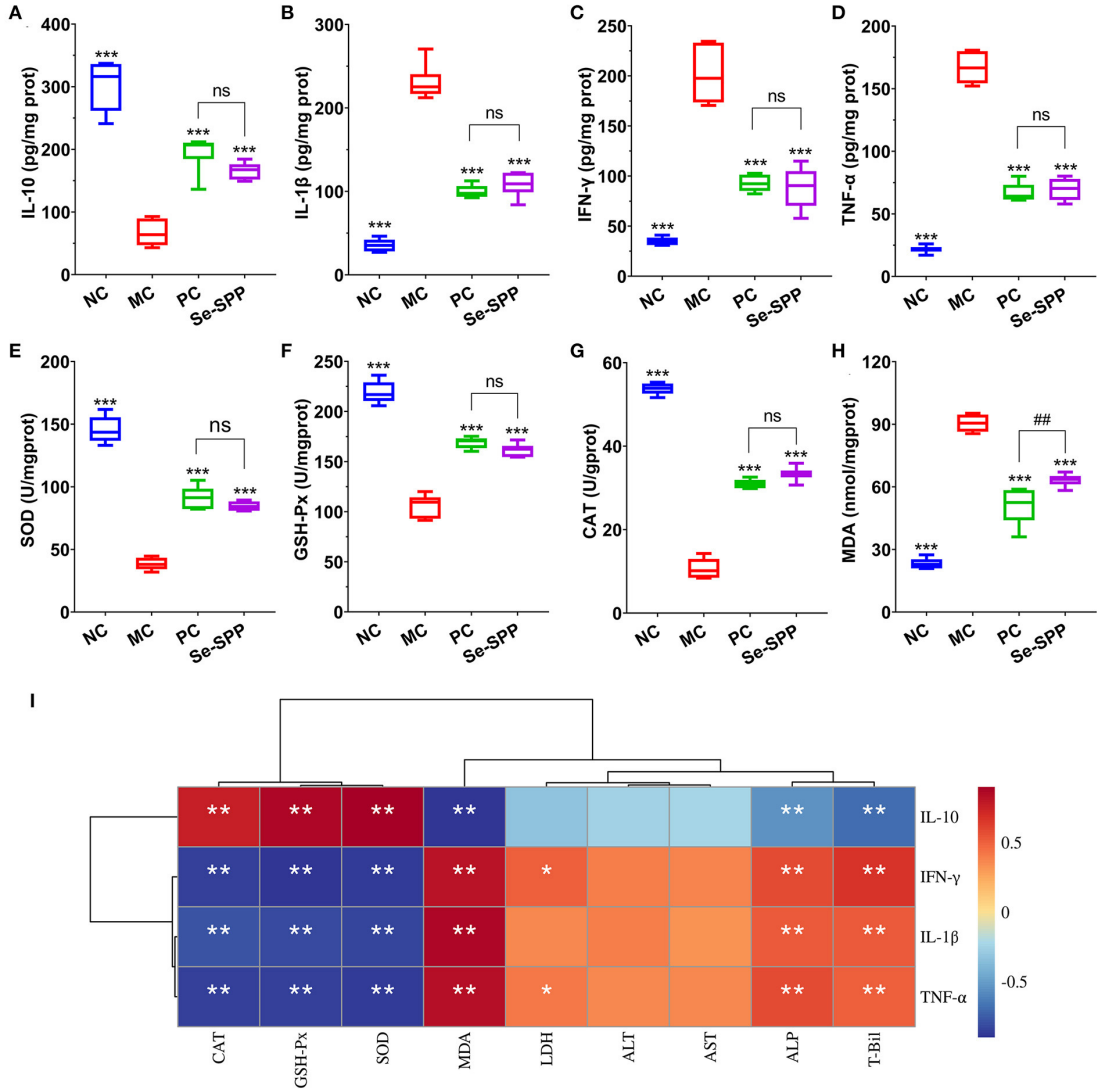
Se-SPP alleviates Cd-induced liver inflammation in mice. Liver TNF-α, IL-1β, IFN-γ, and IL-10 levels **(A–D)**; liver GSH-Px, SOD, CAT, and MDA levels **(E–H)**; and correlation analysis between the inflammatory-related factors and antioxidant indexes **(I)**. NC, normal control; MC, model control; PC, positive control; Se-SPP, selenium-containing polysaccharides from *Spirulina platensis*. TNF-α, tumor necrosis factor; IL-1β, interleukin-1 beta; IFN-γ, interferon-γ; IL-10, interleukin-10; GSH-Px, glutathione peroxidase; SOD, superoxide dismutase; CAT, catalase; MDA, malondialdehyde. Data are expressed as mean ± SEM (*n* = 6). Graph bars marked with ns: not significant,*p < 0.05, **p < 0.01, ***p < 0.001 when compared with MC; ^#^*p* < 0.05, ^##^*p* < 0.01, ^###^*p* < 0.001 when comparing PC with Se-SPP. The colors range from red (positive correlation) to dark blue (negative correlation), and significant correlations are marked with *p < 0.05, **p < 0.01, ***p < 0.001.

The liver inflammatory responses were evaluated by the levels of TNF-α, IL-1β, IFN-γ, and IL-10 in liver tissues using ELISA. As shown in Figures 2A–D, the levels of pro-inflammatory cytokines of hepatic TNF-α, IL-1β, and IFN-γ in the MC group were remarkably increased (*p* < 0.001) compared to those in the NC group, while the content of anti-inflammatory cytokine IL-10 was significantly decreased (*p* < 0.001). However, administration of mice with Na_2_SeO_3_ or Se-SPP was found to obviously suppress (*p* < 0.001) the levels of TNF-α, IL-1β, and IFN-γ but to elevate the level of IL-10 when compared to the MC group. There was no difference in the four inflammatory cytokine levels between the PC and Se-SPP groups (*p* > 0.05).

In order to exhibit the effects of Se-SPP on antioxidant capacity in the liver, the levels of hepatic oxidative stress indicators were determined. As shown in Figures 2E–H, compared with the NC group, the activities of SOD, CAT, and GSH-Px in the liver of the MC group were all downregulated markedly (*p* < 0.001), while upregulating the MDA level (*p* < 0.001). Nonetheless, an obvious increase (*p* < 0.001) in the levels of SOD, CAT, and GSH-Px both in the PC and Se-SPP groups was noted with a concomitant apparent decrease (*p* < 0.001) in the level of MDA as compared to the MC group.

Subsequently, Spearman's correlations between all these inflammatory factors and the antioxidant-related indexes were analyzed. As shown in Figure 2I, the levels of TNF-α, IL-1β, and IFN-γ exhibited significant negative correlations with the levels of CAT, GXH-Px, and SOD (*p* < 0.01) but negative correlations with the level of MDA (*p* < 0.01). Meanwhile, IL-10 exhibited opposite correlations (*p* < 0.01).

### Se-SPP prevents Cd-induced disruption of the intestinal barrier in mice

**Figure 3:**
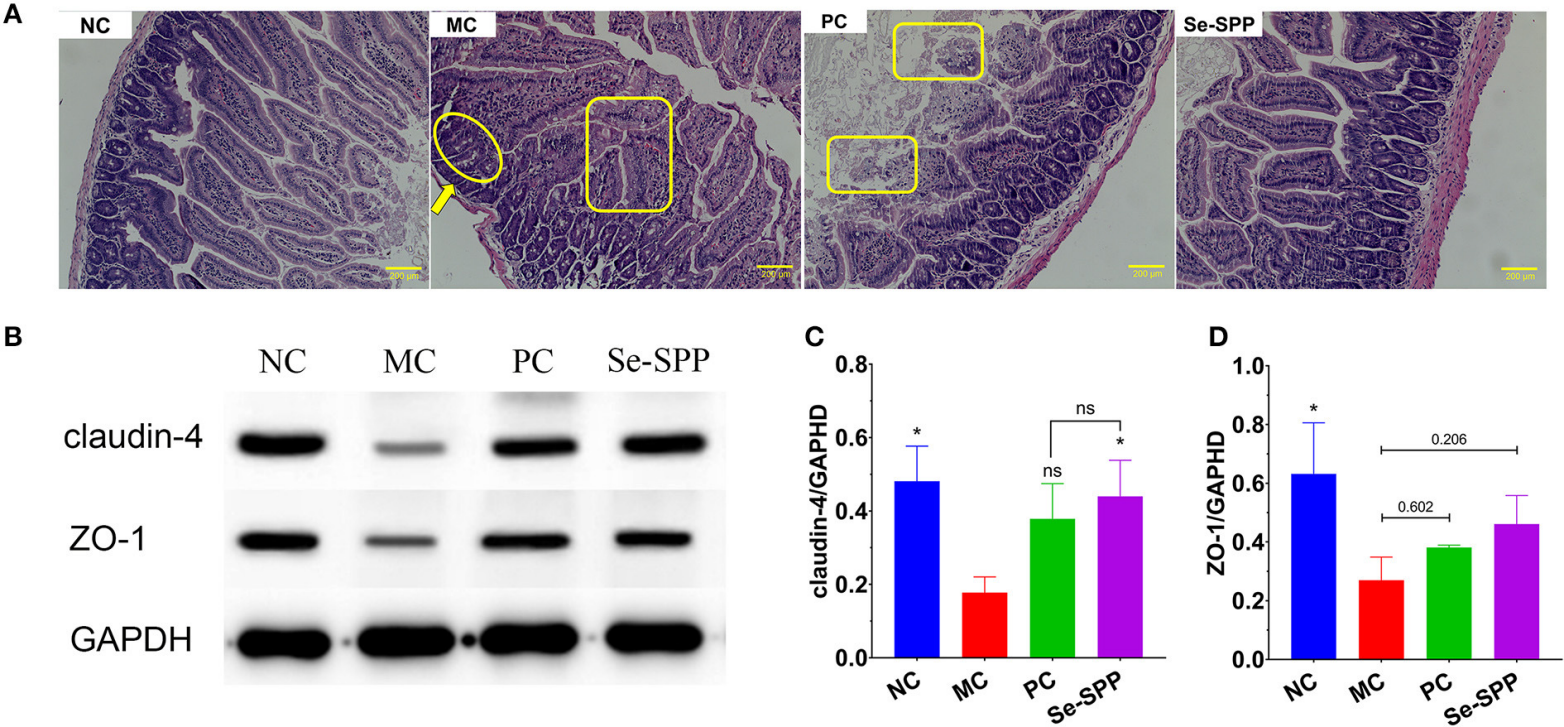
Se-SPP prevents Cd-induced the disruption of the intestinal barrier in mice. Images of the H&E-stained jejunum [**(A)**, 20×]; thinned of muscularis (arrow); fusion or disappearance of villi (circle); disorders in epithelial cells (rectangle). Analysis of tight junctions (TJ) proteins using Western blots **(B–D)**. NC, normal control; MC, model control; PC, positive control; Se-SPP, selenium-containing polysaccharides from *Spirulina platensis*. Data are expressed as mean ± SEM (*n* = 3). Graph bars marked with ns: not significant,*p < 0.05, **p < 0.01, ***p < 0.001, when compared with MC.

The changes in pathological injuries in jejunum tissues could be revealed by H&E staining. As shown in Figure 3A, histological examination of the jejunum tissues of the NC group presented a regular and complete structure with neat villi and intact mucosa, as well as arranged epithelial cells. However, the jejunum tissues in the MC group exhibited obvious pathological changes, including fusion or disappearance of villi, disorders in epithelial cells and thinned of muscularis. With Na_2_SeO_3_ treatment, the PC group exhibited effects on repairing of intestinal damage to a certain extent, but the fusion or loss of villi was still observed. Furthermore, Se-SPP treatment substantially ameliorated the signs of intestinal injury, including relative clear villus structure, tightly packed epithelial cells, and unspoiled muscularis.

The tight junctions (TJs) between epithelial cells are the major components of the intestinal barrier. In order to investigate whether the disruption of the intestinal barrier in Cd toxic mice was restored by Se-SPP, the expressions of two major tight junction proteins (claudin-4 and ZO-1) were determined using Western blot analysis. As shown in Figures 3B–D, claudin-4 and ZO-1 expressions of the jejunum tissues in the MC group were apparently decreased compared to those in the NC group. Se-SPP treatment led to an upregulation of the expression of claudin-4 and ZO-1, which was better than that in the PC group.

### Se-SPP prevents Cd-induced gut dysbiosis in mice

#### Se-SPP reshapes Cd-induced gut microbiota in mice

**Figure 4:**
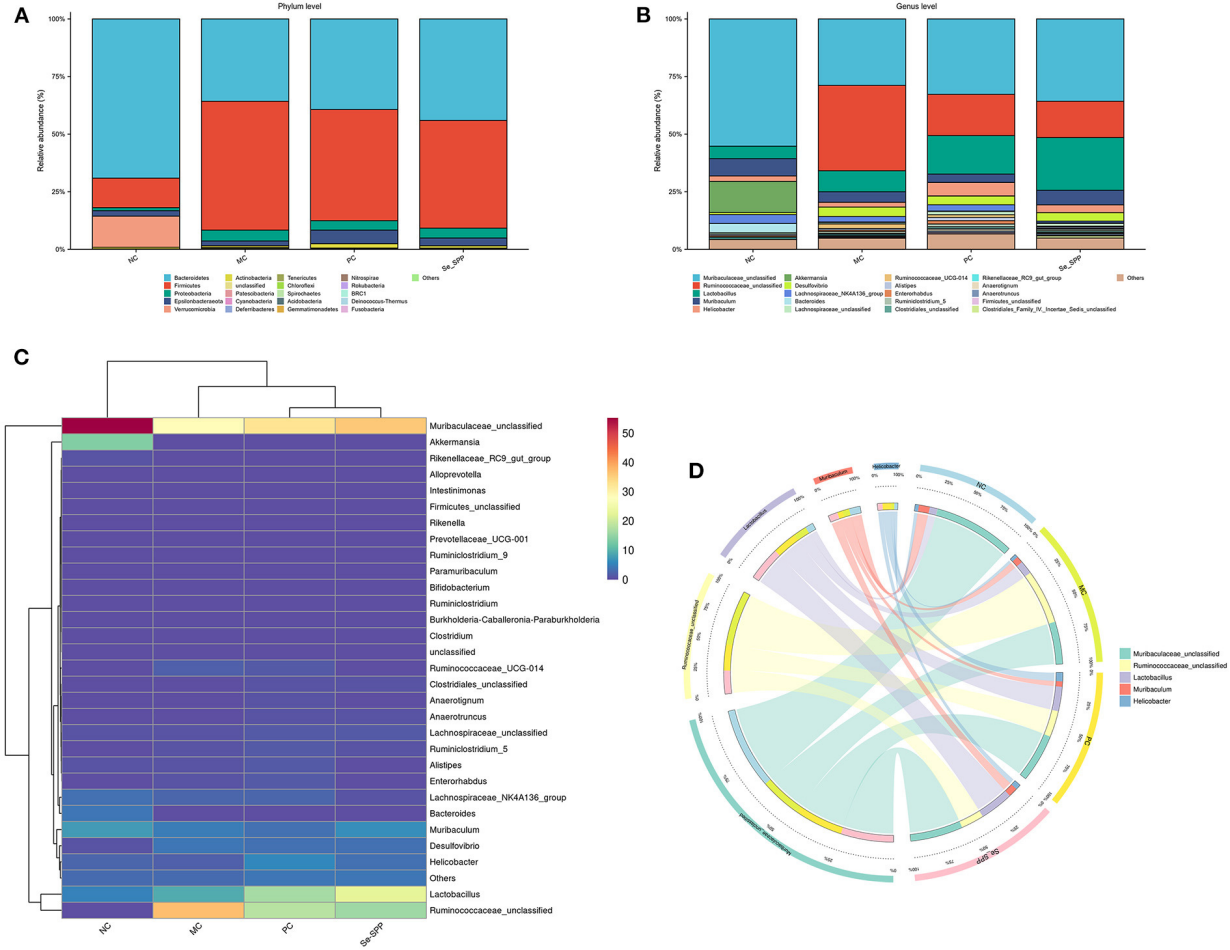
Se-SPP reshapes Cd-induced gut microbiota in mice. Relative abundance at the phylum level **(A)**, relative abundance at the genus level **(B)**, difference analysis of gut microbiota composition at the genus level **(C)**, and community heatmap analysis at the genus level **(D)**. NC, normal control; MC, model control; PC, positive control; Se-SPP, selenium-containing polysaccharides from *Spirulina platensis*. Data are expressed as mean ± SEM (*n* = 3).

The histogram exhibited an apparent difference in the relative abundance of intestinal microbial species among the four groups (NC, MC, PC, and Se-SPP). As shown in Figure 4A, at the level of the phylum, the main bacteria of intestinal microbiomes in the NC group were *Bacteroidetes, Firmicutes*, and *Verrucomicrobia*, accounting for about 95% of the intestinal flora. After Cd^2+^ exposure, the relative abundance of intestinal flora was seriously affected. *Bacteroidetes* and *Firmicutes* were dominant in intestinal microbiomes. Compared with the NC group, CdCl_2_ treatment induced an increase in the relative abundance of *Firmicutes*, as well as decrease in the relative abundances of *Bacteroidetes* and *Verrucomicrobia*. Meanwhile, Na_2_SeO_3_ and Se-SPP therapies improved the relative abundance of *Bacteroidetes*.

At the genus level (Figure 4B), the microbiota was mainly composed of *Muribaculaceae, Ruminococcaceae, Lactobacillus, Muribaculum, Helicobacter, Akkermansia*, and *Desulfovibrio*. The influence of CdCl_2_ substantially increased the relative abundances of multiple genera, including *Ruminococcaceae, Lactobacillus*, and *Desulfovibrio*, when compared to the NC group. Nevertheless, *Muribaculaceae, Muribaculum, Akkermansia*, and *Desulfovibrio* were opposite.

R software was used for cluster analysis of the 30 most abundant genera and draw the heatmap, and the colors were used to reflect the similarity of community composition between groups. A clustering heatmap (Figure 4C) showed an obvious difference in microbiological composition between the normal and Cd-treated mice, and the supplementation of CdCl_2_ altered the microbial composition of the Cd-treated mice. The abundance of *Muribaculaceae* decreased dramatically in the MC group, but its abundance increased after treatment with Na_2_SeO_3_ and Se-SPP. The result of relative abundance of *Ruminococcaceae* was opposite. Consistently, the figure of Circos was comparable to that of the aforementioned (Figure 4D).

#### β-Diversity analysis

**Figure 5:**
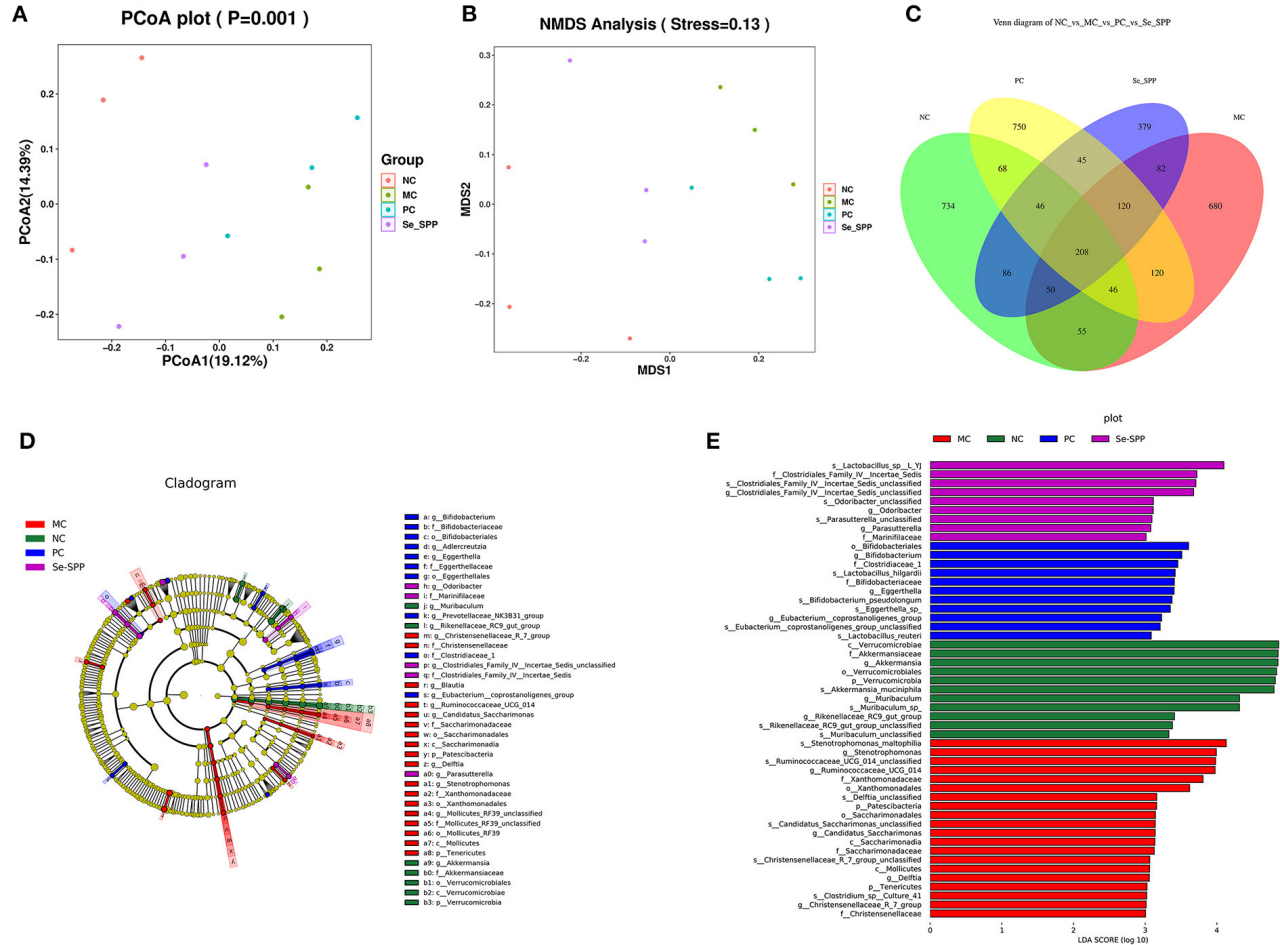
Se-SPP modulates the specific phylotypes of the gut microbiome. β-Diversity representing PCoA and NMDS **(A,B)**, Venn diagram of OTUs **(C)**, and cladogram. From the inside out was the bacterial taxonomic level of phylum, class, order, family, and genus **(D)**, indicator bacteria of intestinal microbiota with LDA scores of 3 or greater in mice **(E)**. NC, normal control; MC, model control; PC, positive control; Se-SPP, selenium-containing polysaccharides from *Spirulina platensis*. Data are expressed as mean ± SEM (*n* = 3).

β-Diversity analysis is used to evaluate the similarity of the community structure between different groups (29). Principal co-ordinate analysis (PCoA) and non-metric multidimensional scaling (NMDS) analysis were used to represent community changes in different samples. As shown in Figure 5A, PCoA1 and PCoA2 accounted for 19.12 and 14.39% of the overall analysis results, respectively. After treatment with CdCl_2_, the MC group was clearly separated from the NC group. In addition, the gut microbiota in mice intervened with Se-SPP was effectively farther away from the MC group and closer to the NC group. The result of NMDS (Figure 5B) was consistent with that of PCoA. These results demonstrated that the stimulation of CdCl_2_ caused an obvious change in the gut microbiota structure in mice, and Se-SPP ameliorated the migration of gut microbiota caused by CdCl_2_. Coherently, the conclusion that Se-SPP intervention modulated Cd-induced gut microbiota dysbiosis in the donor mice could be proved.

#### Se-SPP modulates the specific phylotypes of the gut microbiome

The shared and specific OTUs among different groups were represented by Venn diagrams in Figure 5C. A total of 208 OTUs were shared among the four groups, with the NC, MC, PC, and Se-SPP groups having 734, 680, 750, and 379 of unique OTUs, respectively.

LEfSe analysis was further utilized to identify the statistically significant biomarkers of the gut microbiota in different groups. The cladograms and line discriminant analysis (LDA) score distribution histogram (based on LDA score >3) are shown in Figures 5D,E. A total of 65 taxa in different levels were notably different among the NC, MC, PC, and Se-SPP groups. Furthermore, the number of taxa differentially abundant in the MC group was more than twice that in the NC group, while the number of taxa in the Se-SPP group was the lowest. It can be reflected that Se-SPP modulated some specific gut microbiome in mice caused by CdCl_2_. At the genus level, *Akkermansia, Ruminococcaceae, Bifidobacterium*, and *Clostridiales* were the most dominant differential microbiota in the NC, MC, PC, and Se-SPP groups, respectively. The shift was consistent with the previous analysis.

### Correlation between gut microbiota and inflammation-related parameters

**Figure 6:**
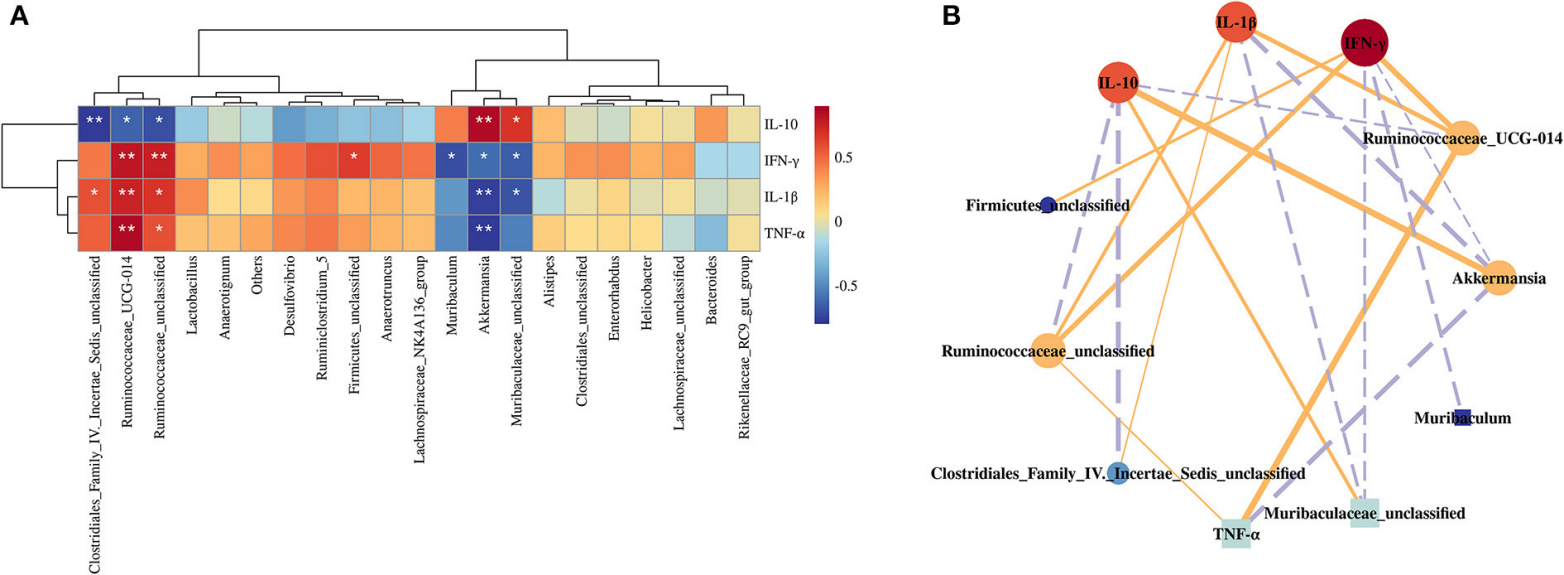
Correlation analysis between the gut microbiota and inflammation-related indexes. Heatmap **(A)** and visual network diagram **(B)**. NC, normal control; MC, model control; PC, positive control; Se-SPP, selenium-containing polysaccharides from *Spirulina platensis*. TNF-α, tumor necrosis factor; IL-1β, interleukin-1 beta; IFN-γ, interferon-γ; IL-10, interleukin-10. The colors range from red (positive correlation) to blue (negative correlation), and significant correlations are marked with *p < 0.05, **p < 0.01, ***p < 0.001.

Spearman's correlation between the gut microbiota (top 30 most abundant gut microbiome) and the inflammation-related parameters was analyzed to identify the specific phylotypes that may contribute to the modulation of Cd-induced injury in the liver. The results showed that about seven specific phylotypes were negatively or positively correlated with inflammation-related traits, respectively (Figure 6). The enrichment of *Ruminococcaceae* indicated positive correlations with the levels of IFN-γ, TNF-α, and IL-1β in the liver but negative correlations with the levels of IL-10. *Akkermansia* was found to negatively correlate with IFN-γ, TNF-α, and IL-1β but positively correlate with the contents of IL-10. Likewise, *Muribaculaceae* was found to negatively correlate with IFN-γ and IL-1β but positively correlate with the contents of IL-10. *Clostridiales* was found to positively correlate with the contents of IL-1β but negatively correlate with IL-10. Furthermore, *Muribaculum* exhibited negative correlations with the levels of IFN-γ, but *Firmicutes* was opposite. Of note, among these genera, *Ruminococcaceae* and *Akkermansia* were highly correlated with the inflammatory factors.

## Discussions

On account of urbanization and industrialization, the degree of environmental pollution has seriously increased. As a non-essential trace element, Cd easily accumulates in animals and humans and exhibits toxic effects on various organs (especially the liver) after acute or chronic intake (30). Some natural polysaccharides and sodium selenite have been utilized to mitigate cadmium-induced organ injury, respectively (31, 32). Although selenium-containing polysaccharides possess better bioavailability compared to inorganic selenium, there is a lack of relevant research on the inhibition of Cd-induced toxicity by it. Our previous work revealed that the treatment with selenium-containing polysaccharides of *Spirulina platensis* (Se-SPP) exhibited significant protection against Cd-induced acute toxicity, but its underlying mechanism lacks study.

The liver have been considered the target organ for toxic effects of cadmium (33). The present study focused on liver injury and inflammation to explore the potential and mechanism of Se-SPP in resisting chronic Cd-induced toxicity. In the present experiment, CdCl_2_ was utilized to build a Cd-induced toxic mice model *in vivo*. Then, the protective effect of Se-SPP against cadmium-induced toxicity was assessed by investigating the liver coefficient, histopathology and serum biochemical indicators, and apoptosis rate, inflammatory markers, and antioxidant enzymes in liver tissues.

After intake of CdCl_2_ in mice, the downregulation of the liver coefficient indicated dehydration and atrophy of the liver due to long-term Cd exposure, which may be caused by pathological changes (34). Some negative pathological changes were observed in hepatic tissues of Cd-induced toxic mice, including inflammatory cell infiltration, hepatocyte karyopyknosis, and disordered hepatic cord. Noticeably, Se-SPP treatment mitigated corresponding lesions in mice compared with the MC group, suggesting that Se-SPP could prevent some of these histopathology changes caused by Cd^2+^.

The level of T-Bil and the activities of ALT, AST, ALP, and LDH are some of the key direct indicators for assessing liver injury in clinical trials. In general, inflammation, necrosis, poisoning, and other damage to the liver lead to increased serum ALT, AST, ALP, T-Bil, and LDH, which indicate enhanced membrane permeability and impaired hepatocytes (35, 36). As evident from this study, the supplementation of CdCl_2_ significantly upregulated the levels of all five biochemical indicators of liver function, while the treatment of Se-SPP downregulated them dramatically. It was reflected that Se-SPP had a protective effect on liver function. Furthermore, liver tissue apoptotic cells appeared when the liver was damaged to a certain degree. The result of the number of hepatic apoptotic cells illustrated that Se-SPP partially decreased Cd-induced hepatic injury in mice by ameliorating apoptosis. In summary, pathological changes, biochemical indicators of liver function, and hepatic apoptotic rate in the mice of the present experiment exhibited the same trends. It was confirmed that Cd exposure resulted in apparent liver damage, indicating that Cd-induced liver injury model *in vivo* was well-established. Similar liver injury phenomena were presented in other related studies (2, 23, 37–39). Simultaneously, Se-SPP possessed significant suppressive effect on cadmium-induced hepatic injury in mice, and the effect was superior than that of inorganic selenium. *Rosa persica* hydroalcoholic extract, caffeic acid phenethyl ester, and chlorogenic acid have been used in associated research studies and exhibited similar results (23, 37, 39).

Undoubtedly, as an exogenous hepatic toxicant, the intake of Cd activated inflammatory response, thereby releasing inflammatory cytokines and aggravating the damage of liver tissues (40). The levels of pro-inflammatory cytokines of hepatic TNF-α, IL-1β, and IFN-γ in mice were remarkably increased (*p* < 0.001) after Cd exposure, while the administration of mice with Na_2_SeO_3_ or Se-SPP was found to obviously suppress (*p* < 0.001) the their levels. The change in the content of anti-inflammatory cytokine IL-10 was opposite. This result confirmed that cadmium exposure caused hepatic inflammatory reactions in the experimental mice, which was consistent with previous reports (25, 41). Meanwhile, the anti-inflammatory activity of Se-SPP has been proved, which supported the results of histopathology changes, biochemical indicators of liver function, and hepatic apoptotic rate. Of note, better results in the Se-SPP group than those in the PC group confirmed that selenium-containing polysaccharides possessed better effects on antagonizing Cd-induced hepatic inflammatory reactions than inorganic selenium alone. Due to its advantages of minimal toxicity and high bioavailability, organic selenium has better effects while avoiding the potential harm of inorganic selenium, the effects of which on inhibiting Cd-toxicity deserve more attention.

Oxidative stress is often considered the main cause of drug- or chemical-induced hepatotoxicity (42). Cd leads to functional and structural impairment in cells by increasing lipid peroxidation (6). Excessive free radicals can suppress the antioxidant capacity of hepatic cells, leading to cell inflammation or apoptosis (40). It can be speculated that one of the mechanisms of Se-SPP in inhibiting cadmium toxicity may be associated with its excellent antioxidant activity. In order to explain the potential mechanism of inhibiting cadmium-induced toxicity, the levels of hepatic oxidative stress indicators were determined. The result demonstrated that exposure of the mice to CdCl_2_ seriously reduced their liver antioxidant capacity. Nonetheless, the problem was alleviated by the supplementation of Na_2_SeO_3_ or Se-SPP. It was revealed that Se-SPP attenuated Cd-induced oxidative stress to resist lipid peroxidation occurred in the liver, which contributed to the amelioration of Cd-induced hepatic damage. Significant correlations among all these inflammatory factors (TNF-α, IL-1β, IFN-γ, and IL-10) and the antioxidant-related indexes (SOD, GSH-Px, CAT, and MDA) provided evidence for the aforementioned speculation, suggesting that antioxidant enzymes may play important roles in inhibiting Cd-induced liver damage and inflammation. In sum, 28-day Se-SPP supplementation in Cd-induced toxic mice significantly mitigated liver pathological damage and inflammation, which is correlated to the upregulation of antioxidant enzyme activity.

Due to the bidirectional relationship between the liver, intestine, and its microbiota, the health of the liver is inseparable from the intestine (12). The intestinal flora is considered an indispensable organ of the host and plays an important role in the degradation of many contaminants such as metals (11). Many research studies have confirmed that gut microbiota is closely associated with heavy metal-induced toxicity. Moreover, the integrity of the intestinal barrier is related to the gut microbiota, which may also in turn affect the absorption of heavy metals (43). Gut barrier function depends on the integrity of gut structure (44). The changes of pathological injuries in jejunum tissue could be revealed that the intestinal barrier of Cd-treated mice could be protected by Se-SPP treatment effectively. Additionally, the assembly of the TJ proteins was vital for the integrity of the intestinal barrier. As two important of TJ proteins, the states of claudin-4 and ZO-1 were critical for maintaining the TJ structure and the formation and maintenance of the barrier function (28, 45). The study has exhibited that wild jujube sarcocarp polysaccharides (WJPs) ameliorated the epithelial barrier by regulating the expressions of four major TJ proteins (occludin, claudin-1, claudin-4, and ZO-1) between epithelial cells (46). Our research showed similar results as the expressions of claudin-4 and ZO-1 in jejunum were promoted by Se-SPP. Taken together, Se-SPP attenuated the disruption of intestinal barrier caused by Cd^2+^, speculating that the effect of Se-SPP on alleviating Cd-induced toxicity in mice may be mediated by the intestinal barrier.

The evidence in the previous study has been proved that a polysaccharide from *Spirulina platensis* (PSP) had significant effects on the gut microbiota in mice, showing the enhancement in abundance of beneficial bacteria (47). It can be speculated that another mechanism of Se-SPP in inhibiting cadmium toxicity may be related to its effect on ameliorating of gut microbiota. Therefore, to explore the effect of Cd^2+^ on gut microbiota and the role of Se-SPP in the regulation of gut microbiota in Cd-treated model mice, 16S rRNA sequencing was used to analyze the composition of gut microbiota. An obvious difference in microbiological composition was found between the normal and Cd-treated mice. *Akkermansia* is considered a new probiotic for degrading mucin and ameliorate the intestinal barrier (48). At the level of the genus, CdCl_2_ treatment remarkably decreased the abundance of *Akkermansia*, implying that Cd treatment caused disruption of the gut barrier. Likewise, the higher relative abundance of *Muribaculaceae* family is correlated with an extended life span (49). As a prevalent gut microbe, *Ruminococcus gnavus* is associated with Crohn's disease (a major type of inflammatory bowel disease), which potently induces inflammatory cytokine (TNF-α) secretion by dendritic cells (50). Interestingly, a similar phenomenon was also found in the present study, showing that the abundance of *Muribaculaceae* decreased dramatically in the MC group compared with the NC group, but its abundance increased after treatment with Se-SPP. The result of relative abundance of *Ruminococcaceae* was opposite. More importantly, Spearman's correlation analysis further revealed that *Ruminococcaceae* and *Akkermansia* were highly correlated with the inflammatory factors. These results revealed that the bacteria *Ruminococcaceae* and *Akkermansia* might play a key role in mediating liver inflammation. Taken together, it can be speculated that the modulation of Se-SPP on the *Ruminococcaceae* population contributed to the improvement of Cd-induced inflammation-related diseases by downregulating TNF-α and IFN-γ in the liver.

## Conclusion

In conclusion, the amelioration of Cd-induced toxicity in mice by Se-SPP might be mediated by inflammatory factors. Se-SPP treatment downregulated the inflammatory cytokines by increasing the antioxidant capacity, and mitigated Cd-induced liver injury in mice. In addition, Se-SPP positively modulated the composition of gut microbiota destroyed by CdCl_2_, which was markedly correlated with its inhibition of inflammatory factors. Hence, Se-SPP alleviated Cd-induced toxicity in mice by inhibiting liver inflammation mediated by gut microbiota. Furthermore, potential key microbes in the efficacy of Se-SPP have been proposed. Present research extended the understanding of the inhibiting effect on Cd-induced toxicity of dietary Se-SPP, but the molecular mechanism requires further investigation.

## Data availability statement

The raw sequence data reported in this paper have been deposited in the Genome Sequence Archive (Genomics, Proteomics & Bioinformatics 2021) in National Genomics Data Center (Nucleic Acids Res 2022), China National Center for Bioinformation/Beijing Institute of Genomics, Chinese Academy of Sciences (GSA: CRA008374) that are publicly accessible at https://ngdc.cncb.ac.cn/gsa.

## Ethics statement

The animal study was reviewed and approved by Animal Ethics Committee of the Guangxi Institute of Chinese Medicine and Pharmaceutical Science (No. 2020110202).

## Author contributions

NZ: conception, writing—original draft, and data curation. HL: conception, data curation, methodology, validation, and software. LY: formal analysis and resources. XX: funding acquisition and resources. ZZ: conception, methodology, investigation, and writing—review and editing. XL: project administration, funding acquisition, and supervision. All authors contributed to the article and approved the submitted version.

## Funding

This work was funded by Guangxi Natural Science Foundation (2020GXNSFAA297038 and 2021GXNSFAA075034) and Guangxi Key Research and Development Program (AB19259010).

## Conflict of interest

The authors declare that the research was conducted in the absence of any commercial or financial relationships that could be construed as a potential conflict of interest.

## Publisher's note

All claims expressed in this article are solely those of the authors and do not necessarily represent those of their affiliated organizations, or those of the publisher, the editors and the reviewers. Any product that may be evaluated in this article, or claim that may be made by its manufacturer, is not guaranteed or endorsed by the publisher.

## Abbreviations

ALP: alkaline phosphatase
ALT: alanine transaminase
AST: aspartate aminotransferase
CAT: catalase
Cd: cadmium
ELISA: enzyme-linked immunosorbent assay
GAPHD: glyceraldehyde-3-phosphate dehydrogenase
GSH: glutathione peroxidases
H&E: hematoxylin and eosin
IFN-γ: interferon-γ
IL-10: interleukin-10
IL-1β: interleukin-1β
LDA: line discriminant analysis
LDH: lactate dehydrogenase
NMDS: on-metric multidimensional scaling
MDA: malondialdehyde
ROS: reactive oxygen species
PCoA: principal co-ordinates analysis
SCFAs: short-chain fatty acids
Se: selenium
SOD: superoxide dismutase
T-Bil: total bilirubin
TNF-α: tumor necrosis factor-α
TUNEL: transferase-mediated dUTP nick-end labeling
WHO: World Health Organization
ZO-1: tight junction protein 1.
